# Exercise is medicine, or exercise with medicine? Comparative effects on Na^+^,K^+^‐ATPase regulation

**DOI:** 10.1113/EP092247

**Published:** 2024-09-30

**Authors:** J. Max Michel, Michael Kamal

**Affiliations:** ^1^ School of Kinesiology Auburn University Auburn Alabama USA; ^2^ Department of Kinesiology, Faculty of Science McMaster University Hamilton Ontario Canada

**Keywords:** digoxin, human/environmental and exercise physiology, potassium transport, sodium–potassium ATPase, sodium transport

1

For over a century, scientists have known that skeletal muscle is highly reliant on sodium (Na^+^) and potassium (K^+^) ions, which fluctuate to change the polarity of the muscle fibre membrane and trigger muscle contractions (McKenna, Renaud, et al., 2024). These ions and their gradients are so critical, in fact, that ion transport is taught in physiology classes from the high school to the doctoral level. The Na^+^,K^+^‐ATPase (NKA) pump maintains these ion gradients and, like many factors in skeletal muscle, is responsive to exercise and pharmacological intervention. In this issue of *Experimental Physiology*, McKenna, Gong, et al. (2024) sought to investigate the genetic regulation of the NKA pump using these two intervention types. To do this, they provided healthy adults with digoxin both at rest and in response to exercise, and then measured the relative change in NKA isoform (α1–3 and β1–3) mRNA expression within their skeletal muscle. Digoxin is an oral pharmaceutical that was primarily used to treat heart failure and atrial fibrillation, but is also capable of inhibiting NKA activity and affecting the expression of NKA‐associated genes (Ren et al., 2021). The results of this study demonstrated that certain α and β subunits were generally sensitive to exercise (α1–3, β3) and that no individual subunit was responsive to DIG administration. Interestingly, when isoform expression was summed and/or pooled, it was observed that total β subunit mRNA expression was impacted by DIG supplementation. Critically, however, no effects of DIG were detected at the protein level, while α2 protein abundance declined at 3 h post‐exercise.

The observed increase in total β subunit mRNA with DIG at baseline is consistent with a prior investigation (Sostaric et al., 2022). The lack of change in α subunits was perhaps unexpected, as these have been previously shown to be up‐ or down‐regulated in response to DIG administration (Wang et al., 2000). It is also striking that while DIG conferred some effect to β subunits, the effects of exercise were much more pronounced throughout all subunits. Although there is certainly excitement about the possibility of a compounding effect in this regard (e.g. DIG + exercise), there is also mechanistic validity to other unintended effects of DIG administration such as enhanced autophagy and apoptosis (Wang et al., 2000). Additionally, while the activation of such molecular pathways is not harmful in the right context, activation of muscle protein breakdown pathways (autophagy) or apoptosis‐related pathways induced by exogenous drug administration could prove to be more of a hindrance than a benefit. Therefore, this warrants further interrogation in skeletal muscle, specifically in the context of weighing benefits of concomitant administration of DIG with exercise versus benefits of exercise alone in NKA gene and protein regulation where autophagy and apoptosis‐related pathways are additionally monitored.

Nonetheless, the findings observed by McKenna et al. are novel and accessible to a wide audience given the widespread knowledge pertaining to Na^+^ and K^+^ ion regulation in skeletal muscle. The information provided herein indicates that exercise, and potentially DIG administration, augments gene expression of NKA transporters and therefore can putatively enhance Na^+^ and K^+^ ion transport. These findings expand the current knowledge base of exercise physiology relating to skeletal muscle contraction, and therefore benefit a wide range of students, physiologists and general practitioners alike.

## AUTHOR CONTRIBUTIONS

Both authors have read and approved the manuscript and agree to be accountable for all aspects of the work in ensuring that questions related to the accuracy or integrity of any part of the work are appropriately investigated and resolved. All persons designated as authors qualify for authorship, and all those who qualify for authorship are listed.

## CONFLICT OF INTEREST

None declared.

## FUNDING INFORMATION

None.
